# The Long Journey to BVD Eradication

**DOI:** 10.3390/pathogens10101292

**Published:** 2021-10-07

**Authors:** Volker Moennig, Matt J. Yarnall

**Affiliations:** 1 Institute of Virology, University of Veterinary Medicine Hannover, 30559 Hannover, Germany; volker.moennig.iR@tiho-hannover.de; 2 Boehringer Ingelheim Vetmedica GmbH, 55218 Ingelheim am Rhein, Germany

**Keywords:** bovine viral diarrhoea virus, BVD, stakeholders, control, voluntary, compulsory, legislation

## Abstract

This paper provides a short review of bovine virus diarrhoea (BVD) control programmes across Europe, with a particular focus on current efforts from a stakeholder perspective. Using outputs gained from a global, virtual congress on BVD control, the theory of the journey from BVD control to possible eradication is enriched with insight from stakeholders representing the major parts of the cattle industry. Current control programmes were presented by Javier Dieguez (Galicia), Neil Shand (England), Neil Paton (Wales), Jenny Purcell (Scotland), Maria Guelbenzu (Ireland), Jörn Gethmann (Germany), and Matthias Schweizer (Switzerland).

## 1. Introduction

The known history of bovine viral diarrhoea (BVD) began in 1946, when a new transmissible diarrhoea was reported [1]. Apart from an acute diarrhoea, no particular damage caused by the infection was known at that time. However, the true nature of the infection was progressively discovered. It took at least 40 years to appreciate the true impact of BVD virus (BVDV) infections. Today, BVD is recognised as one of the most economically important endemic diseases of cattle.

Except for rare outbreaks of virulent BVDV [2], which may kill up to 40% of young cattle, BVD is a stealthy disease that is usually not noticed by the farmer. BVDV is well adapted to cattle and, in most cases, apart from a few days of fever and a loss of appetite, there are no clinical signs of acute infection, which ends after about two to three weeks and leaves the animals immune to reinfection. However, acute infection is always accompanied by transient immunosuppression, which facilitates secondary infections by facultative pathogens and has a massive impact on the fertility of female animals. Only months after the introduction of the infection into the herd, visible effects that the farmer rarely associates with BVD appear. Reproductive failure, e.g., transient infertility, stillbirths, abortions, malformed calves and persistently infected (PI) calves, are typically caused by BVD infection. PI calves are the key epidemiological driver of BVD and result from the transmission of the non-cytopathic BVDV biotype across the placenta of naïve dams during the first 18–120 days of pregnancy. Most die at an early age, but some develop the dramatic clinical picture of fatal mucosal disease, following superinfection with the cytopathic biotype of the same BVDV antigenic makeup [3,4]. About half of PI animals appear clinically normal and their infection can only be detected using laboratory diagnostic methods. Other effects of the infection are reduced herd health, e.g., drop in milk production, clinical mastitis, longer calving intervals, and an increase in respiratory and enteric diseases [5]. Most of the latter effects are attributable to transient immunosuppression by BVD infection.

Many attempts have been made to assess the economic damage inflicted by BVD infections [6,7]. Results are quite heterogeneous depending on study designs, e.g., the type of herd, parameters to calculate losses, and country-specific conditions. However, there is general agreement that BVD causes considerable economic damage and most countries that have performed respective economic analyses have developed programmes for the mitigation and control of BVD. In this paper, the term control is mostly used for ongoing and past programmes rather than eradication, because the latter means the total (100%) and permanent removal of BVD infection, which at present is not always possible.

## 2. Early Control Programmes

Until the late 1980s, it was thought that an effective control of BVD was impossible, both due to the ubiquitous distribution of the infection and a lack of inexpensive and effective laboratory diagnostic assays. In addition–due to the stealthy nature of the disease–the full scope of its economic damage had not yet been appreciated by farmers and veterinarians. Later, the development of fast and inexpensive serological and antigen detection tests [8] based on ELISA techniques provided suitable tools for large scale BVD diagnosis at regional and national levels. However, apart from the technical means to detect BVD infections, awareness of all stakeholders, and financial and legal provisions, are essential for the design and implementation of a control programme. The first regional voluntary control programme was launched in the late 1980s in the state of Lower Saxony in Germany [9]. Since funds were limited and farmers’ awareness in general was still low at that time, control measures were voluntary. The key element of the programme was the search for PI animals using blood samples. Farmers who signed up for the programme received compensation for culled PI animals and laboratory tests were paid for. Soon, it became clear that participating farms—once BVD-free—were facing a high risk of reinfection from infected neighbouring farms, and therefore free vaccination of virus-free herds became part of the programme. For participating farmers, the programme proved quite successful, but due to a lack of wider stakeholder involvement it did not reduce regional BVD prevalence significantly. Due to the long duration of the voluntary approach, costs were very high and no sustainable effect on the BVD prevalence on population level could be achieved.

At the same time Scandinavian countries initiated compulsory BVD control programmes [10]. Vaccination was prohibited and, using serology, infected herds were identified, followed by an intense search for PI animals in positive herds. PI cattle had to be removed and movement restrictions were in place for infected herds. Biosecurity was strict. Some programmes were initially industry-driven, but in the final stages were all supported by legislation. After about 10 years, the Scandinavian countries were basically BVD-free. Cost benefit analyses showed the favourable economic effects of the measures [11]. A few years later, Austria successfully followed the Scandinavian method of BVD control [12]. The Scandinavian approach clearly demonstrated that systematic BVD control is possible and that it has positive effects, e.g., improved herd health and animal welfare, better reproduction results, higher milk yields, better prices for pedigree stock, reduced antibiotic use, and, last but not least, reduced veterinary bills.

## 3. Current Control Programmes

### 3.1. The Role of Stakeholders

During the early control programmes, it became apparent that stakeholder involvement is crucial. This was a primary focus of early BVDzero Congresses, which brought together stakeholders from across the UK and Ireland. The 2021 event was held virtually due to global coronavirus travel limitations, which facilitated engagement on a global level. A specific end-to-end video engagement platform (Livestorm^®^, Woburn, MA, USA) was used to host the live Congress, which consisted of four workshops for the stakeholders to engage in.

Stakeholders registered in advance of the event, which was held on 7 July 2021, with 327 unique registrations from 28 countries. Pre-recorded presentations to provide further context for discussions were available to view from seven speakers, representing seven different programmes on the BVD eradication journey. The speakers were Javier Diéguez, University of Santiago de Compostela, representing the Galician BVD control programme; Neil Shand, Chief Executive of the UK National Beef Association, representing the English BVD eradication programme; Neil Paton, Royal Veterinary College London, representing the Welsh BVD eradication programme; Jenny Purcell, BVD Policy Manager for the Scottish Government, representing the Scottish BVD eradication programme; Maria Guelbenzu, BVD Programme Manager representing the Irish eradication programme; Jörn Gethmann, Friedrich-Loeffler-Institute, representing the German eradication programme; and Matthias Schweizer, University of Bern, representing the Swiss BVD eradication programme. These presentations are available to view online at www.bvdzero.co.uk/blog/bvdzero-congress-2021 (accessed on 3 October 2021)

The four workshops were hosted by speakers representing an eradication programme, and stakeholders contributed to prompted questions in an online visual collaboration platform (Miro^®^, San Francisco, LA, USA). There were 116 stakeholders that participated live for 50% or more of the event. A summary of the responses to questions posed to stakeholders is provided below.

#### 3.1.1. How to Get Started with a Local Control or Eradication Programme?

At the start of a programme, availability of financial support and a database were mentioned as being important; however, they were not as commonly cited as the need for a simple programme linking with other activities or disease control programmes and ensuring maximum stakeholder engagement. Other factors mentioned were ensuring personal engagement with farmers and making use of a competitive element, with clear goals and communication. Some stakeholders with experience of eradication programmes suggested that ear tagging from the start would be beneficial, along with a carrot-or-stick approach to ensure removal of PIs. The take home messages for those starting a BVD control programme were to set realistic targets, communicate well, keep the programme simple and consistent, and back the scheme up with legislation.

#### 3.1.2. Who Are the Stakeholders to Identify for the BVD Eradication Journey and How Can Early Engagement Be Optimised?

Stakeholders span the whole industry, from consumers to data management companies, and from governments to farmers and farming organisations. Reasons for early engagement have included seeing the productivity and health benefits, a sense of public good and pride, as well as pressure from within and outside the industry. Barriers to that early engagement have included upfront costs or perceived costs, prioritising other diseases, and the extra time involved. When asked what resources and capabilities stakeholders could bring to optimise early engagement, there was strong support for government backing, including legislation and funding. A database and transparency of BVD status was also important, along with any other incentives that stakeholders could provide.

#### 3.1.3. How Can Stakeholder Enthusiasm Be Maintained throughout the BVD Eradication Journey?

Congress stakeholders highlighted that clear communication from the start to the end of the programme and evidence of early progress are strong motivators, along with sharing experiences and evidence of benefits of eradication. Pressure from the market, e.g., pedigree buyers, supermarket chains, and consumers, was also a motivator. In contrast, prolonged campaigns and a lack of visible progress were considered the main demotivators. Lack of compliance by other stakeholders and other disease priorities were also considered demotivating. The upfront cost was also highlighted, especially when the impact of the disease is not obvious. Finally, programme complexity was a commonly cited demotivator.

#### 3.1.4. What Does BVD Freedom Look Like and Does Vaccination Have a Place?

Stakeholders emphasised the benefits of BVD control through improved cattle health, welfare, and productivity, including reduced antimicrobial usage. However, there were also some benefits that have historically been less considered, including increased confidence to eradicate or control other diseases and improved farmer mental health. The change to a surveillance system was seen as a potential risk to maintaining BVD freedom since prompt detection of new BVDV outbreaks might not be guaranteed. Industry apathy and non-compliant farms, a false sense of security leading to reduced biosecurity measures, and a naïve national herd were mentioned as major problems for control programmes. The measures proposed to minimise these risks were a continued strict regime of surveillance of herds, rapid tracing of new cases using a database, as well as reliable import controls. This needs to be supported by appropriate legislation and could be combined in areas of increased risk with ongoing vaccination to maintain herd immunity.

### 3.2. Recent Developments

During the last 25 years, the early approaches to BVD control (voluntary and compulsory, with and without vaccination) served as blueprints for a number of regional and national control programmes, that are being applied in several European countries for the mitigation or control of BVD. However, there are some differences between programmes, control tools are being used in several combinations, and stakeholder involvement varies. The BVDzero Congress highlighted the situation in seven European regions and countries. Beyond these programmes, there are control efforts on the way in other European and overseas countries [13].

Variations of the Scandinavian programmes were recently implemented by Switzerland, Germany, Ireland, and Scotland. In countries with high seroprevalence and/or widespread vaccination, direct virus detection was the method of choice rather than serological surveillance. With a seroprevalence of 87%, BVD was endemic in Switzerland when the country launched a massive control effort by testing the national cattle population for BVDV in 2008. In the four years to follow, all newborn calves were tested for the virus and PI prevalence was reduced from 1.3% to 0.02%. Subsequently, after five years of virus detection serological surveillance was introduced using bulk milk or serum samples. A national animal movement database and partial sequencing of viral RNA proved to be of value, particularly in the later stages of the programme, when single PI cattle had to be traced back to their origin and contact herds [14]. Occasionally the cause of seropositivity could be attributed to contacts with small ruminants [15]. Wild ruminants were shown not to be a reservoir for the BVD virus [16]. The latter is in accord with results from other European countries [17]. Today, there are still some, though very few, restricted herds in Switzerland, and it is believed that surveillance must be continued for a long time to come. Weaknesses of the Swiss approach were the cantonal rather than a federal organisation of control, alpine farming, partly improper use of the national database, and inconsequent tracing [18]. Vaccination was prohibited throughout the programme.

In contrast, vaccination as a supplementary control tool was used in Germany, Ireland, and Scotland. Following the first regional voluntary control scheme, most German federal states launched voluntary schemes of their own. Depending on cattle density and the structure of the industry, provisions varied and vaccination was either prohibited or used as an additional control tool. In 2004, BVD became notifiable, and in 2008 national legislation for compulsory BVD control was issued. Three years later, January 2011, it came into force [19]. The search for PI animals was performed using blood samples of adult animals and ear notch samples of newborn calves. The results of laboratory diagnoses were entered into the national cattle database. PI animals had to be removed immediately and trade restrictions were imposed for 40 days. Trade with pregnant animals was suspended until birth and a negative test result of the offspring. Depending on the local epidemiological situation, vaccination could be ordered or prohibited. The programme was very successful. In its course, more than 50 million cattle were tested and 49,000 PI animals were removed. Today’s PI prevalence is 0.005%. At present, the control programme is being revised; vaccination will be prohibited and Germany will apply for the status of “BVD-free”. In hindsight, the control programme was beneficial for the cattle industry compared to no control, but it was expensive. Simulations show that there might still be a risk of a future baseline of PI animals.

The Irish control programme was launched in 2013 and is now in its final stages, with 230 herds under restriction in 2021. The goal is freedom from BVD in 2023. As with similar control programmes, the tail end of a BVD control campaign requires extra strict measures in order to achieve freedom. Increased government-funded vaccination is being used to interrupt new infections in naïve farms that have suffered a “breakdown”. A problem from the beginning was the retention of seemingly “healthy” PI animals. Many farmers tried to fatten and sell these animals. Waning compliance at the end of the programme is also seen as a problem.

Scotland started a voluntary, industry-led BVD control programme in 2010 with funding of screening measures. The attempts were accompanied from the beginning by a BVD-advisory group. In 2013, screening of cattle herds became mandatory, and in the following years screening was intensified and movement restrictions were implemented. Imported breeding animals had to be tested. A database is in use to facilitate the tracking of BVD-positive animals. The gradual tightening of measures over the years has yielded success, and at present about 90% of Scottish breeding herds are BVD-free. Non-breeding herds and imported animals were a challenge for the programme because efforts very strictly focused on breeding herds.

At present, several voluntary schemes are in progress, e.g., in Wales, England, and Galicia in Spain. The latter programme, which is subsidised by the regional government, started in 2004. It is based on serology and PI hunting. In infected herds, all newborn calves are tested and positive animals are subject to movement restriction. Participating herds are classified (0–3) with level 3 being BVD-free. Galicia is a cattle-dense region, and it was noted that progress is slowing over the years. The government intends to extend the programme to all farms in Galicia, which would amount to a compulsory government-led programme.

Another voluntary programme (BVD-Free) was launched in England in 2016. Veterinarians are working with “clusters” of cattle keepers that are participating in the scheme, which is funded in part by the Rural Development Programme. The main incentive for farmers is to achieve the status of BVD-free for their herds. At present, the programme is industry-led, but there are plans to draft legislation for the implementation of a compulsory programme following the voluntary scheme.

In Wales, discussions among stakeholders started in 2011 and testing on farms was launched in 2017. At the time of the Congress, 8601 farms had been screened; 75% of Welsh herds—which underwent BVD testing in conjunction with tuberculosis testing—and 2446 holdings turned out to be BVD positive. A third of the farms have looked for PI animals and 675 animals were identified positive. Half of the PIs stayed on the farm, 25% were slaughtered, and 25% were sold (!). A stakeholder steering group accompanied the voluntary programme. The lack of a database was felt as a critical disadvantage, and thus attempts will be made to link the programme to existing animal databases. Planning with the Welsh Government is in progress to make BVD control compulsory and funding of the programme has been extended to 2023. For a summary of the current BVD programmes presented or discussed at the BVDzero Congress, see Table 1.

## 4. Lessons Learnt 

From the mid-1980s, when first control efforts were launched, until now, progress has been impressive in some countries and painstakingly slow in others. Today, most of Europe still has either no or half-hearted schemes for the control of BVD in place. With respect to the severe economic consequences of the infection, this is shocking and calls for an explanation. The delay in appearance or even lack of dramatic clinical signs is partly responsible for the underestimation of BVD-related risks and the low awareness of farmers. Most important is the failure to assess the real risk posed by PI cattle. PI animals are the unique feature of BVD pathogenesis. They represent a most potent reservoir for infections of susceptible animals, and they play a pivotal role in the maintenance of the infection in cattle populations. Control efforts that do not identify and radically remove PI animals are doomed to fail. This requirement is most challenging. Lack of compliance or inconsequent action are the main reasons for ineffective or futile control schemes. Considering these facts, it becomes clear that BVD is more difficult to control than an infectious disease with clear, overt clinical signs and without permanent virus-shedders. Key components of each BVD control programme must be as follows:

- Identification of BVD-infected herds;
- Identification and timely removal of PI animals (test and cull);
- Movement restrictions for cattle with no clear status;
- Quarantine rules for incoming animals;
- Biosecurity;
- Thorough tracing of infections, preferably using a database;
- Depending on the control strategy, vaccination may be prohibited or applied as an additional control tool (immunisation of female cattle to prevent a new generation of PI calves);
- Classification of animals and herds according to their BVD status;
- Solid funding and clear compensation rules;
- Intense liaisons with stakeholders.

In light of these conditions, voluntary programmes are especially difficult to manage, because they require a high degree of awareness, motivation, and compliance from stakeholders, in particular participating farmers. However, many new programmes are the result of democratic discussions among stakeholders. Instead of drafting a straightforward and strict control programme that was proven successful in other countries, many compromises are made for seemingly economic reasons that jeopardise control efforts from the beginning. Typical examples are unclear or no regulations concerning the fate of known PI cattle, lax or no provisions for movement restrictions and biosecurity, and a lack of quarantine rules. Such deficiencies guarantee a long duration of the programme, high costs, and poor performance, if not failure. Successful voluntary programmes would require a strong resolve for success from all stakeholders. Ideally, a steering group of key stakeholders should facilitate communication with farmers and coordinate education and control measures. Throughout the programme, efforts must be monitored, and data management is important. In some countries, existing cattle databases were successfully used to support BVD control. Stumbling blocks are the poor visibility of the damage done in many herds with endemic BVD, the complexity of the programme, a long duration, and the high costs of the schemes. Clear and simple messages as well as peer pressure may help to maintain motivation and enthusiasm of stakeholders. The trusted veterinarian plays an important role in this context. Two particularly critical aspects of voluntary approaches are the challenge of recruiting 100% of farmers to sign up, and the serious threat posed to naïve participating herds by infected neighbouring farms. Most if not all voluntary programmes have a long duration, are expensive, and progress relatively slowly. Consequently, the tail ends of these programmes often become compulsory government-driven schemes.

In contrast, compulsory legislation-led programmes of systematic BVD control so far have been very efficient, as has been demonstrated in several European countries. As with all programmes, compulsory schemes also require professional communication and a high level of awareness and motivation from all stakeholders. Their main advantage is that all farmers are obliged to participate. However, just as with voluntary approaches, they also need the full support of stakeholders, and a lack of compliance can cause serious setbacks and delays.

Next to the complexity of the control process, a lack of trade incentives in the past is also to blame for the slow progress in Europe. For a long time in BVD control efforts, there were no international trade regulations in place with respect to BVD. The Terrestrial Code of the OIE still does not have a chapter on BVD control and corresponding trade regulations and, in the EU, the only BVD-related rules were confined to germinal products. This has changed with the introduction of the European Animal Health Law [20]. For the first time, BVD is listed as a category C disease for optional control and in the Commission Delegated Regulation [21] provisions on control programmes and the granting and maintenance of disease-free status are laid out. These are similar to the “old” rules for listed diseases, e.g., bovine leucosis, with respect to articles 9 and 10 [22]. With respect to laboratory diagnosis of BVD, current EU legislation refers to the “OIE Manual of Diagnostic Tests and Vaccines for Terrestrial Animals” [23]. BVD control of establishments, Member States, or zones is described in detail in Part VI of Regulation (EU) 2020/689 [21]. The Commission may authorise BVD control programmes for Member States or zones and it will acknowledge the status of free from BVD. The competent authorities may grant or withdraw the status of free from BVD to cattle establishments. With respect to trade with cattle, additional guarantees must be given by the dispatching partner depending on the status of the destination partner. However, since there is a certain heterogeneity in the design of control programmes, the probability of freedom from infection must be assessed in order to secure trade [24]. In Member States or zones that are free from BVD, vaccination is prohibited.

## 5. Conclusions

Over the last three decades, a clear path towards successful BVD control has emerged. Provided there is political will, strong stakeholder support, and the application of proven methods, control of this economically important disease should no longer be a problem. With the new legislative background, it can be expected that BVD control in Europe will gain momentum in the next few years.

## Figures and Tables

**Table 1:** A summary of current BVD eradication programmes presented or discussed at the BVDzero Congress 2021.

Eradication Programme	Year Started	Current Stage (V/ M) ^1^	Surveillance Diagnostic Method	Key Performance Indicators (Varies by Programme)	Possibility to Vaccinate	Legislation	Database
Galicia	2004	V	Serology (check test)	0.25% (PI prevalence of purchased cattle)	Yes	No	No
England	2016	V	Virus detection (ear tag) or serology (check test)	40.9% (breeding cattle tested)	Yes	No	Yes
Wales	2017	V	Serology (check test)	75% (breeding herds tested)	Yes	No	No
Northern Ireland	2013 (2016 M)	M	Virus detection (ear tag)	0.31% (PI prevalence)	Yes	Yes: movement restrictions encouraging PI removal	Yes
Scotland	2010 (2013 M)	M	Virus detection (ear tag) or serology (check test)	90% (breeding herds negative)	Yes	Yes: movement restrictions encouraging PI removal	Yes
Ireland	2012 (2013 M)	M	Virus detection (ear tag)	0.03% (PI prevalence)	Yes	Yes: movement restrictions encouraging PI removal	Yes
Germany	2011	M	Virus detection (blood samples and ear tag)	0.005% (PI prevalence)	Yes (regulated by legislation, restrictions from 2021)	Yes: movement restrictions and PI removal	Yes
Switzerland	2008	M	Virus detection (blood samples and ear tag) followed by antibody (bulk milk and check test)	99.58% (herds negative)	No	Yes: movement restrictions and PI removal	Yes

^1^ V = Voluntary, M = Mandatory.

## Data Availability

Stakeholder contributions provided during the BVDzero Congress are available from matt.yarnall@boehringer-ingelheim.com.
